# Membrane current series monitoring: essential reduction of data points to finite number of stable parameters

**DOI:** 10.3389/fncom.2014.00120

**Published:** 2014-09-26

**Authors:** Raoul R. Nigmatullin, Rashid A. Giniatullin, Andrei I. Skorinkin

**Affiliations:** ^1^Theoretical Physics Department, Institute of Physics, Kazan Federal UniversityKazan, Russia; ^2^Department of Neurobiology, A.I. Virtanen Institute, University of Eastern FinlandKuopio, Finland; ^3^Laboratory of Neurobiology, Department of Physiology, Kazan Federal UniversityKazan, Russia; ^4^Department of Radioelectronics, Institute of Physics, Kazan Federal UniversityKazan, Russia; ^5^Department of Biophysics of Synaptic Processes, Kazan Institute of Biochemistry and Biophysics Russian Academy of SciencesKazan, Russia; ^6^Department of Bioinformatics, Institute of InformaticsKazan, Russia

**Keywords:** noise analysis, detrended fluctuation analysis, fluctuation spectroscopy based on beta-distribution, sequence of the ranged amplitudes, membrane currents of neurons

## Abstract

In traditional studies of changes in cell membrane potential or trans-membrane currents a large part of the recorded data presents “a pure noise.” This noise results mainly from the random openings of membrane ionic channels. Different types of stationary or non-stationary noise analysis have been used in electrophysiological experiments for identification of channels kinetic states. But these methods have a limited power and often cannot answer to the main question of the experimental study: do external factors induce a significant change of channels kinetics? A new method suggested in the current study is based on the scaling properties of the beta-distribution function that allows reducing the series containing 200,000 and more data points to analysis of only 10–20 stable parameters. The following clusterization using the generalized Pearson correlation function allows taking into account the influence of an external factor and combine/separate different parameters of interest into a statistical cluster considering the influential parameter. This method which we call BRC (Beta distribution-Reduction-Clusterization) opens new possibilities in creation of a largely reduced database while extracting specific fingerprints of the long-term series. The BRC method was validated using patch clamp current recordings containing 250,000 data points obtained from the living cells and from open tip electrode. The numerical distinction between these two series in terms of the reduced parameters was obtained.

## Introduction

During electrophysiological studies it is common to record rather long tracks of signals. These signals are registered as temporal variations of cell membrane potential or trans-membrane currents induced by the opening of some ligand- or voltage-gated or even chaotic ionic channels. Usually the principal aim of such a study is the registration of some macroscopic signals—evoked or spontaneous—and the change of parameters of these signals characterizes the total effect of some actions that are located in the experimental object. But a large part of the record forms a so-called “empty track” containing a “pure noise” only. It is well known that this noise reflects mainly the result of random openings of transmembrane ionic channels. Different types of stationary or non-stationary noise analysis have been used for identification of these channels' states (Neher and Sakmann, 1976; Sigworth, 1980, 1985, 1986; Läuger, 1985; Traynelisa and Jaramilloa, 1998; Alvarez et al., 2002; Venkataramanan and Sigworth, 2002).

Unfortunately, these methods have not come into widespread use among physiologists since they often cannot answer the main question of the study: If this drug or this change of environment state induces the reliable change of channels condition or not?

**Figure 1:**
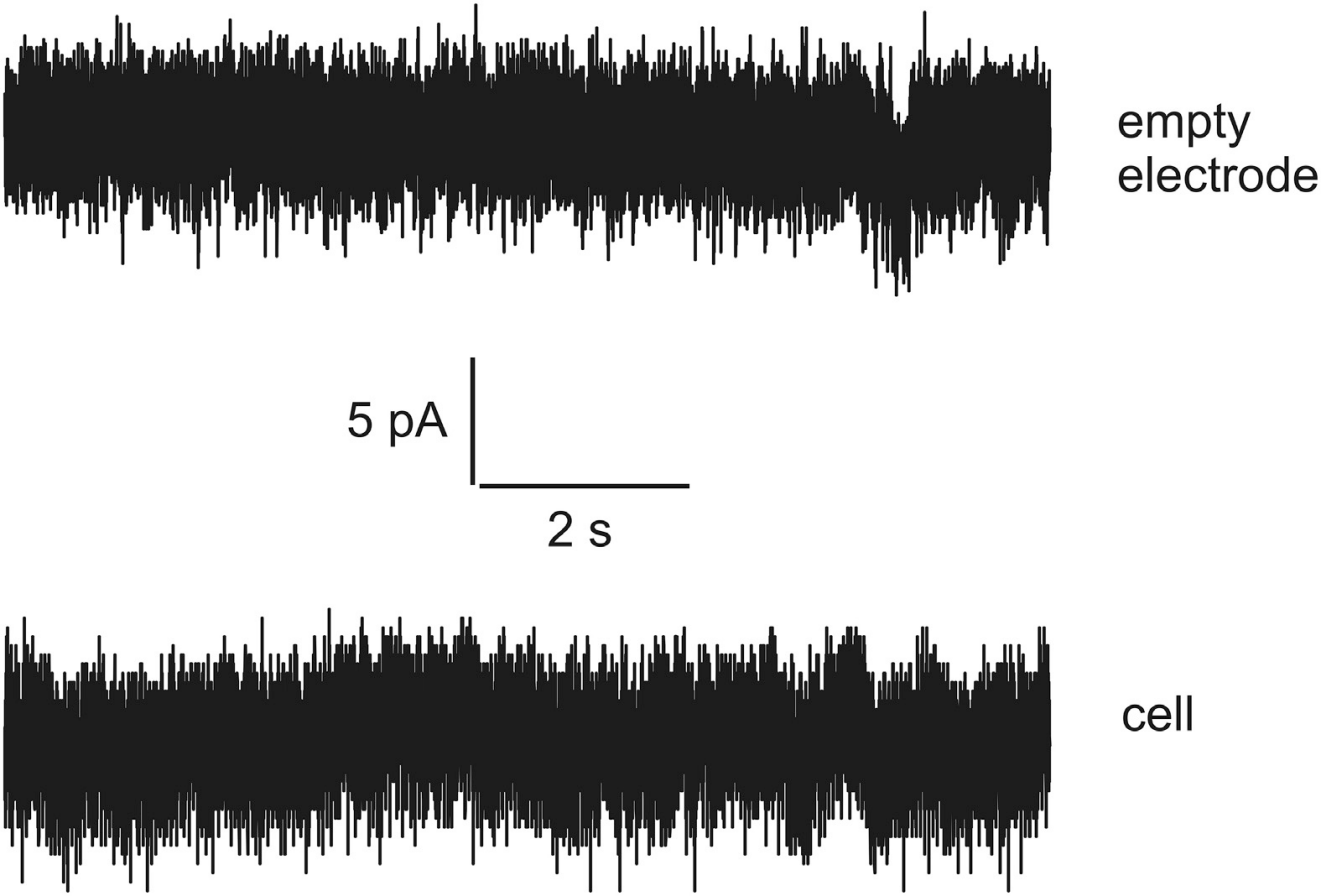
**The examples of currents recorded for empty electrode (top) and in a living cell (bottom)**. Note the high similarity of the tracks.

Thus, there is an urgent task to develop a special language that can be compact and reliable in order to describe accurately very long current streams (long-time series) with hidden signals and noise in terms of a finite and statistically understandable set of reduced parameters. In this paper we want to show *how* to develop this special language based on an example of the analysis of signals recorded in rat's spinal cord slices. Besides this problem we want to show how to detect the presence of the biological object inside the experimental set. For this purpose we also recorded data representing the dependence of the current vs. time when the biological object is absent. Examples of currents recorded in a living cell and with empty electrodes are shown in Figure 1. It is well noticeable that these two signals are apparently very similar. Even though generally distinguishable by an experienced observer the reliability of these differences cannot be numerically evaluated without some special analytic methods.

To the authors' best knowledge one method is basically suitable for quantitative analysis of the different long-time series. This method was introduced by Peng et al. (1994) and nowadays it is known as detrended fluctuation analysis (DFA). It was well described in literature by their creators (Ossadnik et al., 1994; Peng et al., 1995) and found its application in analysis of biomedical (Penzel et al., 2003; Jospin et al., 2007; Burr et al., 2008) and other (Hausdorff et al., 1995, 1996) data. But it is necessary to note that the DFA algorithm works well only for certain types of non-stationary time series (especially having slowly varying trends), it is not designed to handle all possible non-stationarities in real-world data. This algorithm was *not* free also from uncontrollable errors that are associated with approximate fitting of detrended fluctuations by the segments of straight lines or by the parabolic or high order polynomials (Kantelhardt et al., 2001). The final straight line with power-law exponent α_DFA_ is obtained as a slope in a double-log scale as a result of the fitting procedure and contains the fitting error that depends also on the type of segmentation of the initial series considered. These uncontrollable errors (usually they are not properly analyzed in the literature) can lead to different results in calculation of the desired value of the α_DFA_ and other associated fitting parameters in analysis of the *same* long-time series.

A technique, called scale-dependent Lyapunov exponent (SDLE, see Gao et al., 2006, 2012b, 2013; Hu et al., 2010), provides a more comprehensive characterization of complex time series. Some of DFA's limitations have been overcome recently as well by using a new method called adaptive fractal analysis (AFA, see Gao et al., 2010, 2011, 2012a; Riley et al., 2012; Kuznetsov et al., 2013). AFA has been shown to be able to determine global trends, remove noise, perform fractal analysis and multiscale decomposition and present data as a curve. However, new tools could be developed specifically designed to show and estimate even mild differences between two long time series.

Thus, it would be desirable to have a new method with “high resolution” (10–20 significant parameters) to distinguish more accurately the experimental data and effect of treatments. In this paper we demonstrate such method based on some invariant properties of the beta-distribution function; furthermore this method admits a procedure that controls the error in each stage of its application. From our point of view the effectiveness of new approach is based on the monotone behavior of the primary fitting parameters that admit the secondary fit. This peculiarity allows compressing initial fitting parameters with the help of the secondary fit and present initial data set in more compact form.

The four fitting parameters (*A*, *B*, α, β) of beta-distribution can be interpreted and used for quantitative *reading* of fluctuations arising on different scales of the long-time series considered. In previous papers (Nigmatullin, 2010; Nigmatullin et al., 2012) based on the principle of the strong correlation of random sequences it was shown that the cumulative (integral) curve obtained from the sequence of the ranged amplitudes (SRA) can be described with high accuracy by means of the beta-distribution function. In other words, any *detrended* random sequence being transformed to the SRA (when all amplitudes of the initial sequence are sorted out and located in the descending order *y*_1_ > *y*_2_ > … > *y_N_*) after elimination of its mean value and subsequent integration, forms a bell-like curve *J*(*x*) that can be fit (with controllable relative error) by the function:

(1) $$J(x)≅Jb(x)=A{\left(x-x_{0}\right)}^{\alpha }{\left(x_{N}-x\right)}^{\beta }+B.$$

Here the limiting values *x*_0_ < *x_N_* define the ends of the location interval of the random sequence considered. In many cases the parameters *x*_0_, *x_N_* are known. Other quantitative parameters (*A*, *B*, α, β) should be found from the fitting procedure of the function *J*(*x*) to the curve *Jb*(*x*). The power-law exponents (α, β) reflect the *fractal* properties of the random sequence considered and the presence of the memory that is expressed in the behavior of the corresponding SRAs. The criterion for the verification of the presence of memory in two random sequences which are compared is as follows. If one SRA being plotted with respect to another one forms a curve close to a straight line then these two random curves are defined as a having a relative memory and can be considered as being *strongly correlated*. This important property allows transforming any segment of a random sequence to a beta-distribution function and “read” this segment in terms of four unknown fitting parameters (*A*, *B*, α, β). Such transformation from 30 to 50 or even more initial points belonging to a random sequence can be read in terms of these four parameters only. This allows us to suggest a new type of spectroscopy based on some scaling properties of the beta-distribution. This transformation is called Fluctuation Spectroscopy based on Beta-Distribution (FSBD). In general we suggest a method which we call BRC (Beta distribution-Reduction-Clusterization). The basic problem that is solved in this paper by using the BRC method can be formulated as follows: *Is it possible to suggest a reliable method with controllable error that has a wide range of applicability and which has a flexible small set (10–20) of statistically understandable parameters for quantitative characterization of the differences between long-time series?*

## Materials and methods

### Preparation of spinal cord slices

Ten- to Twenty-days-old Wistar rats were deeply anesthetized with diethyl ether and killed by decapitation. After laminectomy, the spinal cord was excised, and immediately immersed in cold (0 ÷ 4°C) artificial cerebrospinal fluid containing (in mM): 126 NaCl, 26 NaHCO_3_, 2.5 KCl, 1.25 NaH_2_PO_4_, 2 CaCl_2_, 2 MgCl_2_, and 10 glucose (bubbled with 95% O_2_ and 5% CO_2_; pH 7.3; 310 mOsm measured). Several transverse slices (250-μm thick) were prepared from the lumbosacral enlargement (L4-6) with a vibratome (VT1000S, Leica, Nussloch, Germany).

### Whole-cell recordings

Slices were transferred to a recording chamber (300 ÷ 400 μl volume) and continuously superfused with oxygenated artificial cerebrospinal fluid at 3 ml/min and 22 ÷ 24°C. Interneurons were visualized with an upright interference contrast microscope and a × 40 water immersion objective (Axioscope FS, Carl Zeiss, Oberkochen, Germany). Patch-pipettes (tip resistance, 5 ÷ 7 MΩ) were prepared by a puller (Flaming-Brown P97; Sutter, Novato, CA, USA) from borosilicate capillaries and were filled with intracellular solution consisting of (in mM: potassium gluconate 140, NaCl 10, MgCl_2_ 3, HEPES 10, EGTA 11; pH 7.3 adjusted with KOH; 300 mOsm measured).

Interneurons were voltage-clamped at −65 mV in the whole-cell configuration after obtaining GV seals (usually not less than 2 GV) by means of a patch-clamp amplifier (Axopatch 200B; Molecular Devices, Sunnyvale, CA, USA). Compensation of capacitance (Cm) and series resistance (Rs) was achieved with the inbuilt circuitry of the amplifier. Series resistance was compensated by 40 ÷ 70% and did not change appreciably from the beginning to the end of the experiments, indicating stable recording conditions. The tracks used for comparison were recorded by the immersion of filled patch-pipettes in artificial cerebrospinal fluid; the patch-pipettes were voltage-clamped at −65 mV too.

Then all data were sampled at 10 kHz and stored on-line with a PC using the pClamp 10.0/Clampex 10.0 software package (Molecular Devices).

### Scaling properties of the beta-distribution and description of the treatment procedure

In this section we want to demonstrate the scaling properties of Expression (1). We subject *x*, *x*_0_ and *x_N_* in Expression (1) to the following scaling transformations, keeping the power-law exponents α and β invariable: *x* = ξ · *x*′ + *b*. *x*_0_ = ξ · *x*′_0_ + *b*, *x_N_* = ξ · *x′_N_* + *b*, which gives the following beta transformation:

(2) $$Jb(x)\to Jb(x^{\prime })=A^{\prime }{\left(x^{\prime }-{x^{\prime }}_{0}\right)}^{\alpha }\cdot {\left({x^{\prime }}_{N}-x^{\prime }\right)}^{\beta },$$

where *A*' = *A* · ξ^(α + β)^. This is the accurate mathematical result that follows from the scaling transformation of the initial coordinates.

In order to have a simple criterion for comparison of the two beta-distributions let us calculate the values of two extreme points *x*, *x*′ belonging to the functions *Jb*(*x*) and *Jb*(*x*′) respectively.

(3) $$\begin{array}{l} \bar{x}=w_{1}x_{0}+w_{2}x_{N},{\bar{x}}^{\prime }=w_{1}{x^{\prime }}_{0}+w_{2}{x^{\prime }}_{N},\\ w_{1}=\frac{\beta }{\alpha +\beta }=\frac{x_{N}-\bar{x}}{\Delta },w_{2}=1-w_{1},\Delta =x_{N}-x_{0},\Delta^{\prime }=\frac{1}{\xi }\Delta ,\\ \bar{H}=Jb\left(\bar{x}\right)=Aw_{1}^{\beta }w_{2}^{\alpha }\Delta^{\alpha \;+\;\beta }+B,\\ {\bar{H}}^{\prime }=Jb\left({\bar{x}}^{\prime }\right)=A^{\prime }w_{1}^{\beta }w_{2}^{\alpha }{\left(\Delta^{\prime }\right)}^{\alpha \;+\;\beta }+B,\\ {\bar{H}}^{\prime }=Jb({\bar{x}}^{\prime })=Aw_{1}^{\beta }w_{2}^{\alpha }\xi^{\alpha \;+\;\beta }{\left(\frac{1}{\xi }\right)}^{\alpha \;+\;\beta }+B\equiv \bar{H}. \end{array}$$

From Expressions (3) it follows that for the scaling transformation (2) the heights *H*, *H*′ of the extreme points of the two bell-like distributions at the fixed values of the power-law exponents α and β and parameter *B* should *coincide* with each other.

Besides this criterion it is necessary to take into account the scaling relationship between the heights *H*, *H*′. If two power-law exponents α and β are subjected to the scaling transformation at the fixed value of the length Δ = *x_N_* − *x*_0_:

(4) $$\alpha^{\prime }=\theta \alpha ,\;\beta^{\prime }=\theta \beta ,$$

then simple manipulations lead to the second scaling relationship:

(5) $$\frac{{\bar{H}}^{\prime }}{A^{\prime }}={\left(\frac{\bar{H}}{A}\right)}^{\theta }.$$

Here the amplitudes *A* and *A*' are defined by relationships (1) and (2), respectively. The consideration of the scaling properties of the beta-distribution allows one to suggest the following two steps.

*Step 1*. This step includes the formation of the sequence of the range amplitudes (SRA) when all amplitudes located on the fixed length Δ = *x_N_* – *x*_0_ are ordered in descending order *y*_1_(*x*_0_) > *y*_2_ > … > *y*(*x_N_*).

*Step* 2. Numerical integration of the SRA with respect to its mean value and subsequent fit to the function (1).

**Figure 2:**
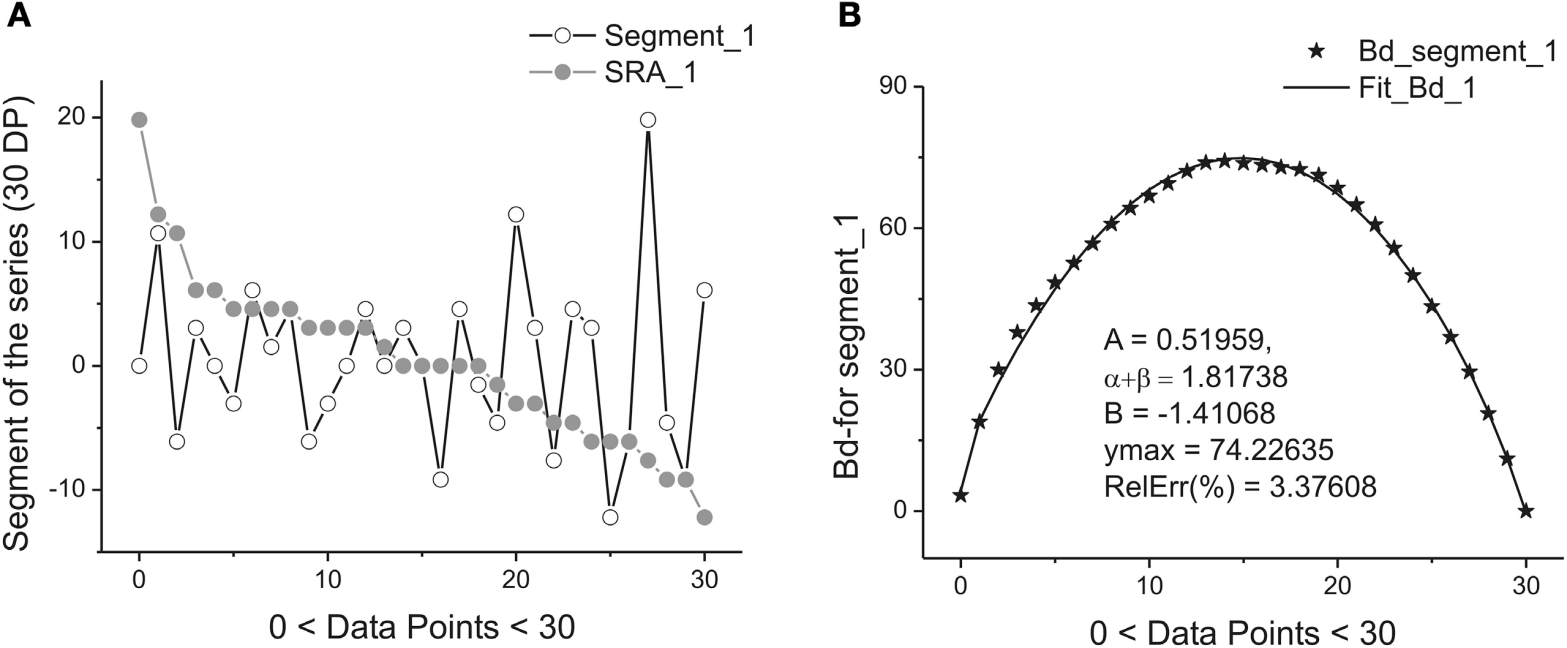
**Example of one segment (marked as Segment_1) containing 30 points. (A)** The sequence of the ranged amplitudes (SRA) given in descending order and marked by gray stars. On vertical axes the values of the current are given in picoampers. **(B)** The bell-like curve (marked by crossed stars) obtained by integration from SRA_1 (shown on the previous **A** by gray stars) and its fit marked by the bold solid line. The fitting parameters of this curve are given inside of this figure. As it follows from this figure 30 data points are sufficient for providing the acceptable fit with the value of the relative error close to 3.5%.

Figure 2 illustrates this transformation which is realized after application of these two steps.

Each sub-segment having equal length Δ is transformed to its SRA (Figure 2A) in Step 1, and the integration of the SRAs with respect to its subtracted mean value gives finally the desired bell-like curve that can be fit to Expression (1) in Step 2. Mathematically these two steps correspondingly are expressed as:

(6a) $$\begin{array}{l} SRA(y(x_{j}))=sort(y(x_{j}))\to \Delta SRA(y(x_{j}))\\ \;=SRA(y(x_{j}))-\frac{1}{\Delta }\sum_{j\;=\;1}^{\Delta }SRA(y(x_{j}))\\ \;\equiv SRA(y(x_{j}))-\langle \ldots \rangle . \end{array}$$

Here the integer index *j* (*j* = 1, 2, …, *N*) numerates the number of data points in the fixed segment Δ = *x_N_* – *x*_0_ containing initially 30–50 data points.

(6b) $$\begin{array}{l} J(x_{j})=J(x_{j})+\frac{1}{2}\left(x_{j}-x_{j-1}\right)\cdot (\Delta SRA(y(x_{j}))\\ \;+\;\Delta SRA(y(x_{j}))),\;J_{0}=0. \end{array}$$

Figure 2 demonstrate the realization of these two steps [with the usage of Expression (6)] on a short segment belonging to the membrane current initial time segment (containing 250,000 data points). We should notice that the mean value <…> of the chosen segment should be subtracted and the integration procedure [the last row in (6)] should be realized with the help of the trapezoid method. As a result of calculation of Expression (6) we obtain the desired bell-like curve *J*(*x_j_*).

Figure 2B shows the quality of the fitting of the bell-like curve obtained to the beta-distribution. In order to have the value of the relative error:

(7) $$\begin{array}{l} \text{ Re}lErr=\left(\frac{stdev(J(x)-Jb(x))}{mean(J(x))}\right)\cdot 100\%,\\ where\;stdev(f(x))={\left[\frac{1}{N_{\Delta }}\sum_{j\;=\;1}^{N_{\Delta }}{\left(f\left(x_{j}\right)-mean\left(f(x)\right)\right)}^{2}\right]}^{1/2},\\ \;mean\left(f(x)\right)=\frac{1}{N_{\Delta }}\sum_{j\;=\;1}^{N_{\Delta }}f(x_{j}), \end{array}$$

to be limited to a few percentages (2–5)% we should choose the length of the minimal segment Δ_min_ of the initial series containing initially 30–50 data points. In Expression (7) the value *N*_Δ_ defines the number of data points that enters in the segment of the length Δ. Thus, the first reduction criterion should be written as:

(8) $$\Delta_{\min}\cdot \xi^{k}=N_{\text{total}}$$

Here the scaling parameter ξ has the same meaning as in Expression (2).

This requirement allows one to consider the long-time series containing the total number of data points (*j* = 1, 2, …, *N*_total_) in terms of the reduced parameters of the beta-distribution (*A*, *B*, α, β) depending on parameter *k*. Further it is convenient to rewrite condition (8) in the following form changing the numeration of the current parameter *k*:

(9) $$\Delta_{k}=\frac{N_{\text{total}}}{\xi^{K\;+\;1\;-\;k}},\;k=1,2,\ldots ,K+1,$$

where [in comparison with (8)] the value Δ_1_ should coincide with the minimal value 30 < Δ_min_ < 50 giving the condition for finding the limiting value of K (the total number of segments is equaled to K + 1). In the opposite case, the value Δ_K_ + 1 should give the maximal length coinciding with the value *N*_total_. As a result of this reduction procedure one can transform *N*_total_ data points to 4.(*K* + 1) parameters. But this step is *not* sufficient. If the functions *A_k_*, *B_k_*, (α + β)_k_ have monotonic behavior one can realize further reduction to the *primary* set of the fitting parameters describing these functions.

Now it is necessary to explain why the sum of the parameters (α + β) is selected instead of considering each-power law exponent separately. This selection is based on the comparison of these exponents with the single power-law exponent α_DFA_ figuring as the basic parameter in the DFA. It is easy to see that relationship α + β = 1 with α ≈ β ≈ 0.5 (for this case beta-distribution looks like a semicircle) corresponds to a distribution with the absence of power-law correlations in the time series. From another side it gives for α_*DFA*_ = 0.5. Comparison with these two power-law exponents leads us to the following approximate expression:

(10) $$\alpha_{DFA}≅\frac{1}{2}\left(\alpha +\beta \right).$$

One can notice also that Expression (10) does not contradict other well-known power-law exponents (Hausdorff et al., 1995; Burr et al., 2008) β_*f*_ = 2α_*DFA*_ − 1 that is used for description of the power-law spectrum *S*(*f*) ~ *f*^−*p*^_*f*_ and decay of autocorrelation function *C*(*t*) = 〈*x_i_x*_*i*+1_〉 ~ *t*^−1^ with γ = 2 − 2 α_*DFA*_. From the requirements (β_*f*_, γ > 0) it follows that:

(11) $$1\leq \left(\alpha +\beta \right)=2\alpha_{DFA}\;\leq \;2.$$

We want to stress here that this requirement is *approximate* and can serve as an indication for division of long-time series with fractal structure (because it does not contradict with well-known inequalities) known before from series with self-similar structure.

The left-hand inequality follows from the requirement β_*f*_ > 0 and does not contradict with numerical results obtained in other papers (Penzel et al., 2003; Jospin et al., 2007; Burr et al., 2008). We should also note that the equality (α + β) = 2 corresponds to a *uniform* amplitude distribution. The uniform distribution leads to the degeneration of the corresponding SRA to a straight line (Nigmatullin, 2010). The beta-distribution in this case is described by a parabolic curve. If one of the power-law exponent (say α → 0) then the position of extreme point *x* → *x*_0_. Because of normalization *w*_1_ + *w*_2_ = 1 β → 1. This statement is valid also in the opposite case when α → 1, β → 0. So, the last relationship (11) can be considered as a *specific fractal* test in our further calculations. Here we should also note that in practical applications the existence of the interval 0 < α + β < 1 and inequality α + β > 2 also are *possible*. For the first case, for small values of α and β the beta-distribution degenerates to a rectangle-like curve. In the second case the values of the derivatives on the ends (*x*_0_, *x_N_*) of the beta-distribution have zero values. These two cases correspond to degeneration of the fractal properties of the time-series analyzed. The verification of relationship (11) on the Weierstrass-Mandelbrot function that represents itself the self-affine function (see its definition in Feder, 1988) confirms the relationship (11). So, for practical purposes it is useful to work with the combination of (α + β).

**Figure 3:**
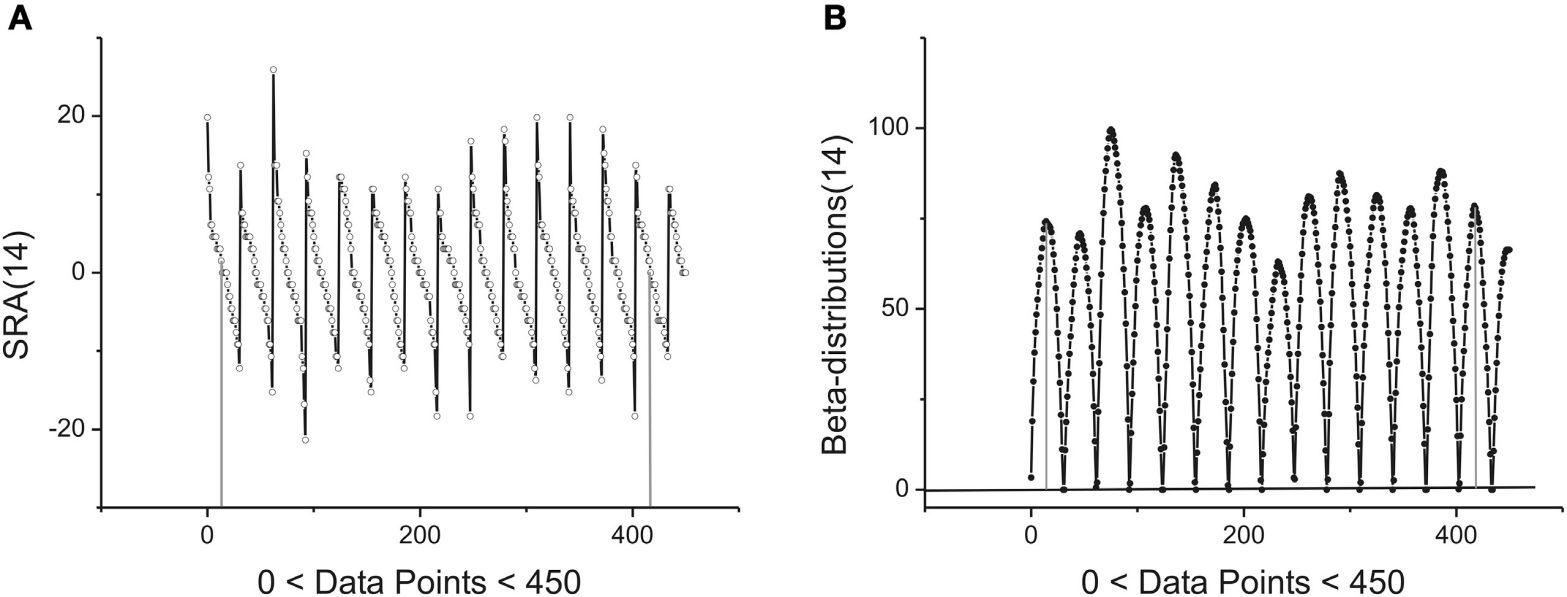
**Example of first 14 segments, each segment contains 30 points. (A)** The first 14 SRAs calculated for the large-time membrane current sequence containing in total 250,000 data points. After elimination of their mean values (two limiting of them are shown by vertical gray lines) and subsequent integration one can obtain a family of the bell-like curves. They are shown below. **(B)** The first 14 beta-distributions obtained by numerical integration from the SRAs given of the previous panel. For the total sequence having 250,000 data points we have in general 8333 distributions of such kind. Two limiting heights are marked by solid gray lines.

The statistical and geometrical meaning of other parameters entering to (1) can be explained as follows. The value of the amplitude *A* together with the height *H* of the beta-distribution is associated with intensity of the fluctuations analyzed. As one can see from Figure 3A the angle of the SRA slope counted off from zero point (after elimination of its mean value) is proportional to the height of the corresponding fluctuation that is expressed in the form of a beta-distribution in Figure 3B. If this angle approaches the vertical axis, the height of the distribution becomes large. In the opposite case when this angle tends to zero the height of the distribution is small. See Figure 3B where the first 14 beta-distributions are shown. The measure of asymmetry can be connected with parameters *B* and the values of weight factors *w*_1,2_ that are defined by Expression (3). The value *w*_1_ = 0.5 corresponds to the complete symmetry of the distribution in the horizontal direction. Any shift of this parameter to the left- (*w*_1_ < 0.5) or to the right-hand side (*w*_1_ > 0.5) reflects the *horizontal asymmetry* of the distribution. A small asymmetry of this distribution in vertical direction is controlled by the parameter *B*.

*Step* 3. After selection of the scaling parameter ξ and the limiting value *K* from Expression (9) one can obtain a family of bell-like curves that can be fitted to Expression (1). The calculated fitting parameters *A*_*k*_, α_*k*_, β_*k*_, *B_k_*, *k* = 1, 2, …, *K* + 1 from Expression (1) are obtained. The set of these bell-like curves and the corresponding fitting parameters forms the total fluctuation spectrum based on the beta-distribution (FSBD). Each part of this FSBD contains the corresponding beta-distribution:

(12) $$Jb_{k}(x_{j})=A_{k}{\left(x_{j}-x_{0,k}\right)}^{\alpha_{k}}{\left(x_{N,k}-x_{j}\right)}^{\beta_{k}}+B_{k}.$$

**Figure 4:**
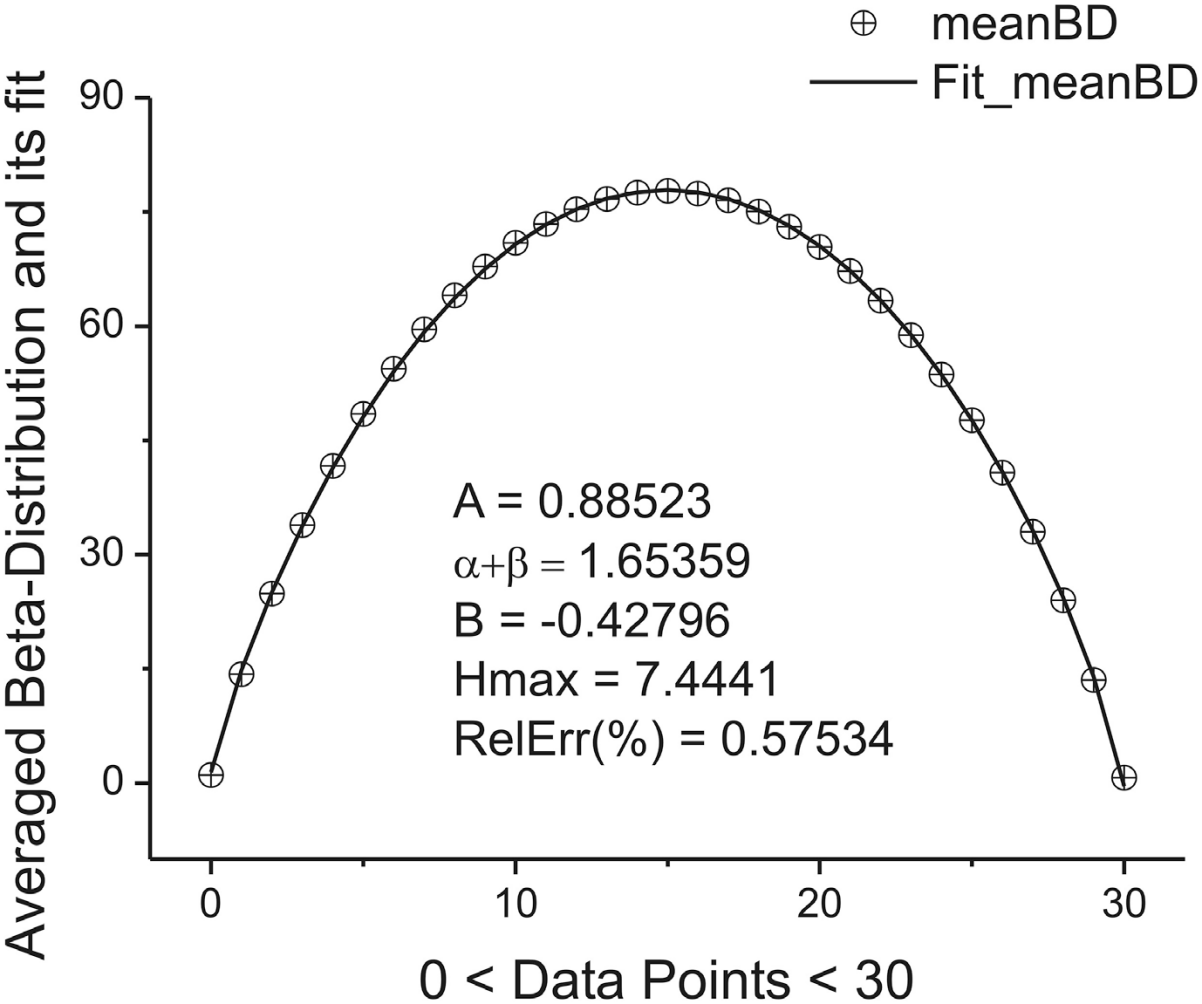
**The averaged beta-distribution (averaged with the usage of Expression (13) for the given *NBd*_1_ = *N_total_*/Δ_1_ = 8333)**. The fitting parameters are shown inside of this figure.

*Step* 4. In order to subject them to the scale-invariant properties described above it is necessary to average this family of distributions and consider only one weighted distribution:

(13) $$\begin{array}{l} \langle Jb_{k}(x_{j})\rangle =\frac{1}{NBd_{k}}\sum_{j\;=\;1}^{NBd_{k}}Jb_{k}(x_{j}),\;j=1,2,\ldots ,NBd_{k},\\ \;NBd_{k}=\frac{N_{total}}{\Delta_{k}}, \end{array}$$

located in the given interval Δ_*k*_. Here the parameter *NBd_k_* coincides with number of beta-distributions calculated for the given *k*. Figure 4 shows the averaged beta-distribution obtained for the cell number 3. If *N*_total_ = 250,000 then from condition (9) at the given Δ_1_ = 32 and ξ = 2 we obtain that *K* = 13. So, the total number of beta-distributions *NBd*_1_ = *N*_total_/Δ_1_ = 8333. The first 14 distributions belonging to this family is shown in Figure 3B.

*Step* 5. Further calculations are reduced to the analysis of the functional dependencies *A_k_*, α_*k*_, β_*k*_, *B_k_*, *k* = 1, 2, …, *K* + 1 with respect to the variable *k*. We define them as the *primary* fitting parameters characterizing the averaged distribution (13). Further analysis shows that the amplitude *A_k_* has monotonic behavior and can be described by a simple exponential behavior:

(14) $$\langle A_{k}\rangle =A_{1}\cdot \exp(\lambda_{a}\cdot k)+A_{0}.$$

Preliminary calculations show that this monotonic behavior is conserved for the long-time series without any trend. The presence of trend distorts this behavior.

**Figure 5:**
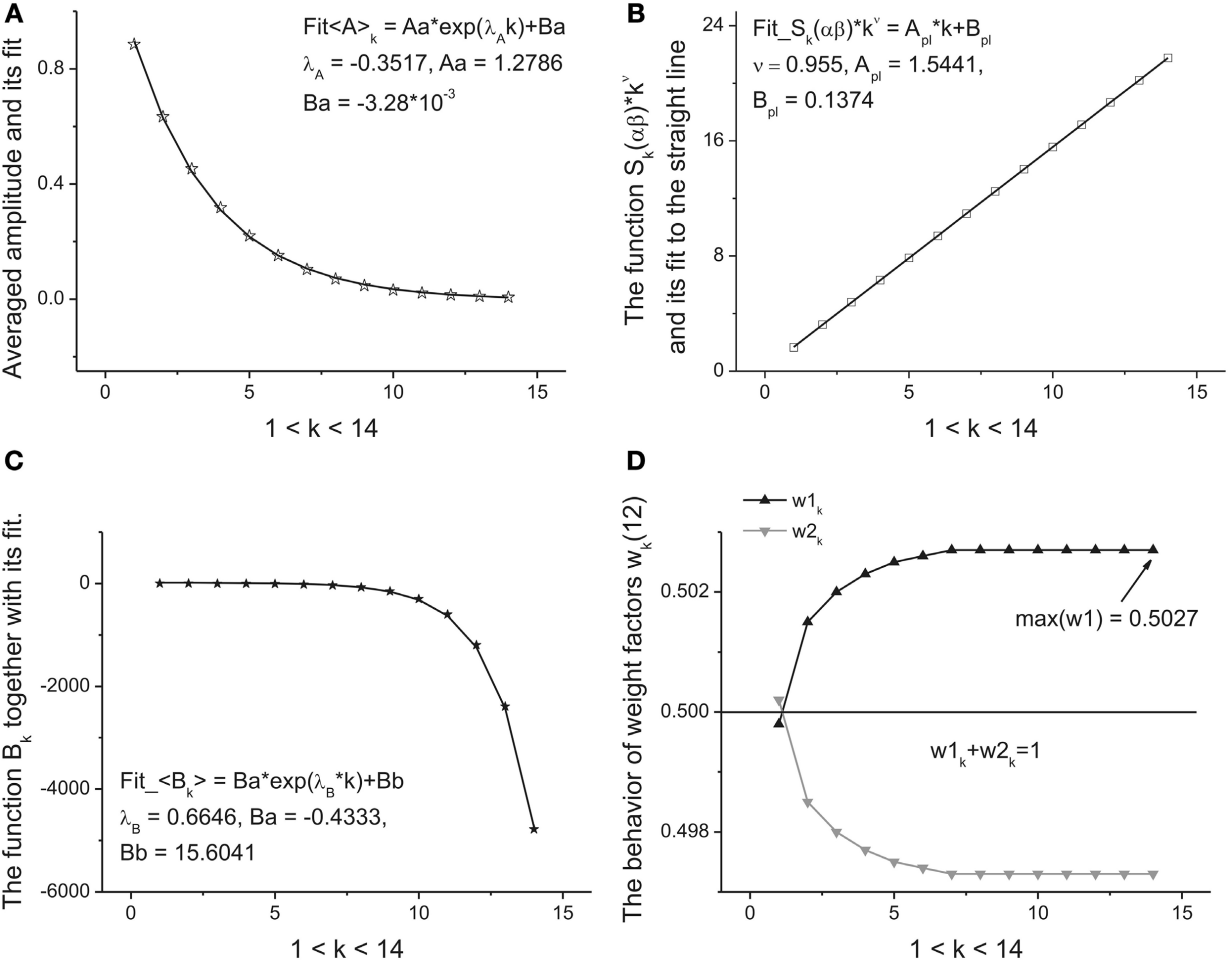
**The fitting curves of four parameters. (A)** The fit of the amplitude obtained for the averaged beta-distributions for different values of *k*. See Expression (14) for details. The fitting parameters of the exponential function are given above of this figure. **(B)** The fit of the function *S_k_*(αβ) = (α + β)*_k_* defined by Expression (15). Being separated by the power-law exponent with ν = 0.955 it represents the perfect straight line. The slope and intercept of this line are given above of this figure. **(C)** The fit of the monotonic decreasing function <*B_k_*> defined by Expression (15). The three fitting parameters of this function can be added to the previous ones for characterization of the given long-time series. **(D)** The behavior of the weight factors with respect to the parameter *k*. As the significant factor characterizing the behavior of the long-time sequence we use the maximal value max(*w*_1_) = 0.5027. So, from analysis of the Figure 4 and in this figure we can extract 10 *primary* fitting parameters.

This dependence follows after substitution of Expression (9) in relationship (2) for the amplitudes. The perfect fit of this monotone curve is shown in Figure 5A. Other dependencies are not so simple but nevertheless they can be identified from simple power-law and exponential hypothesis with the help of the eigen-coordinates (ECs) method (Baleanu et al., 2011; Ciurea et al., 2011). The dependences <(α + β)_k_ > ≡ *S*_k_(αβ) and <*B_k_*> have also monotonic character and can be fitted by means of two simple functions:

(15) $$\begin{array}{l} S_{k}\left(\alpha \beta \right)\cdot k^{\nu }=A_{pl}\cdot k+B_{pl},\\ \;\langle B_{k}\rangle =B_{1}\cdot \exp\left(\lambda_{B}\cdot k\right)+B_{0} \end{array}$$

These functions are shown, respectively, in Figures 5B,C. So, finally we obtain 10 fitting parameters that can be combined with 9 parameters figuring in Expressions (14) and (15) [λ_a_, *A*_1_, *A*_0_], [ν, *A_pl_*, *B_pl_*], [λ_*B*_, *B*_1_, *B*_0_] and the limiting value of parameter *w*_1_,_K+1_. The behavior of this weight factor is shown in Figure 5D.

**Figure 6:**
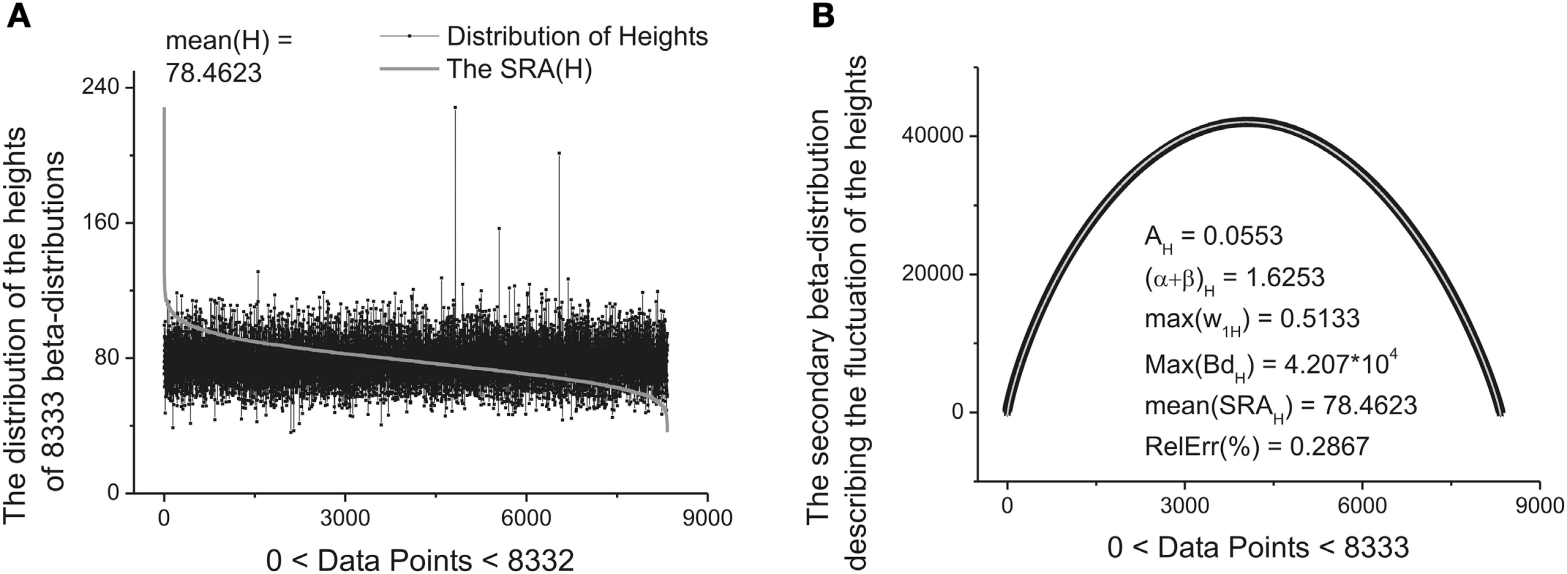
**The distribution of the heights of 8333 beta-distributions (when each distribution occupies only 30 data points). (A)** Subtracting the mean value of this distribution [mean(H) = 78.4623] one can obtain the bell-like curve again. This curve can be fitted it to the secondary beta-distribution corresponding to the distribution of fluctuations of the heights. **(B)** The fit to beta-distribution function corresponding to fluctuations of the heights. The five fitting parameters of this distribution (shown inside of this figure) can be used as the statistically significant parameters for characterizing of the long-time series considered.

**Figure 7:**
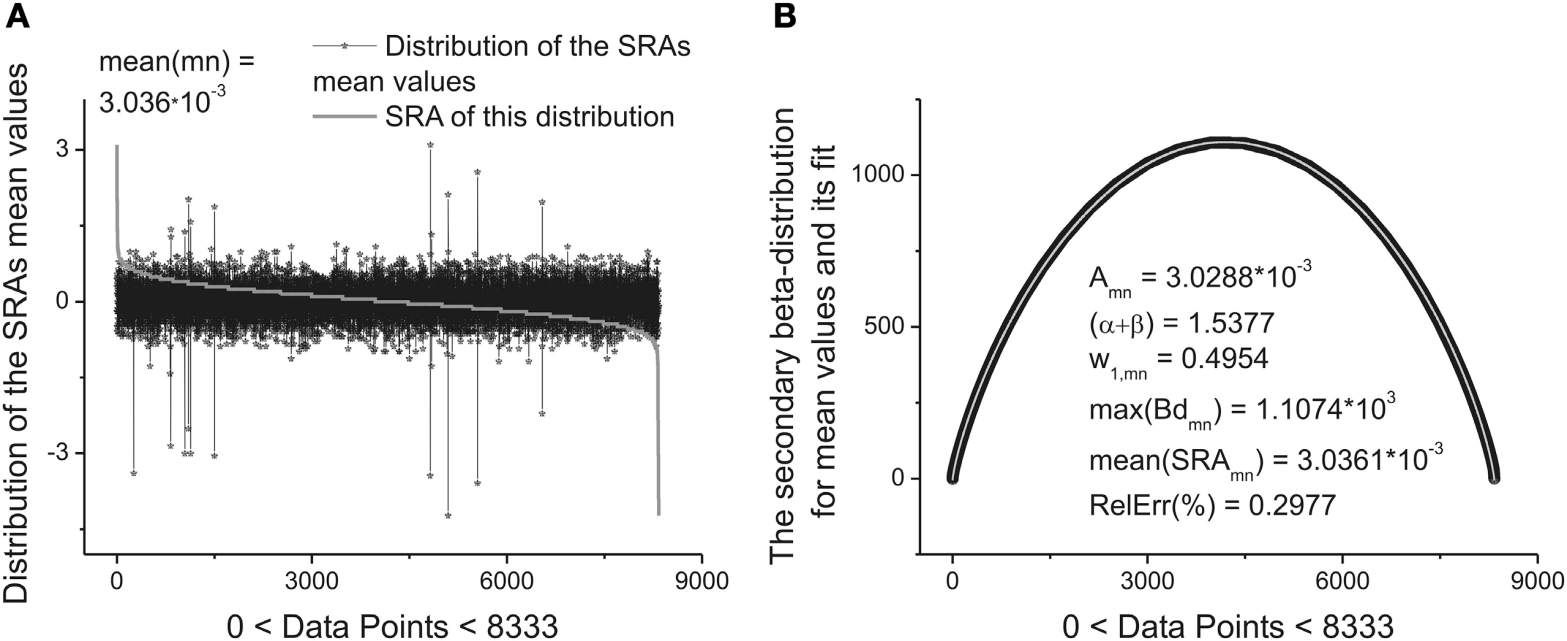
**The distribution of the mean values of 8333 beta-distributions (when each distribution occupies only 30 data points.) that were calculated in the initial analysis. (A)** Subtracting the mean value of this distribution (mean(mn) = 3.036.10^−3^) one can obtain again the bell-like curve. This curve can be fitted it to the secondary beta-distribution corresponding to the distribution of mean values. **(B)** The fit to beta-distribution function corresponding to the fluctuations of the mean values. This information was lost at the preliminary analysis. The five fitting parameters of this distribution (shown inside of this figure) can be used as the statistically significant parameters for characterizing of the long-time series considered. So, in the results of this complete analysis one can obtain 20 statistically significant parameters that can be used for the detailed classification of the long-time series containing 2.5.10^5^ ÷ 10^6^ data points.

These ten parameters can be used as the *primary* set of the fitting parameters for creation of a specific “fingerprint” of the long-time series considered. The idea of clusterization of these parameters is discussed in Results Section. Further analysis shows that the distribution of the heights and mean values of the SRAs obtained for the family of distributions at Δ_1_ also forms two other different beta-distributions. These distributions are *important* also for clusterization purposes because initially the information about the secondary distribution of the heights of the initially formed beta-distributions family and mean values of the corresponding SRA were *not* taken into account. The distributions of the heights and mean values together with their beta-distributions are shown in Figures 6, 7, correspondingly. After fitting of these two distributions one can obtain in addition 5 significant parameters characterizing each beta-distribution separately.

(16) $$\begin{array}{l} \;\left[A_{H},{\left(\alpha +\beta \right)}_{H},w_{1,H},\;\max(Bd_{H}),\;mean(SRA_{H})\right],\\ \left[A_{mn},{\left(\alpha +\beta \right)}_{mn},w_{1,mn},\max(Bd_{mn}),\;mean(SRA_{mn})\right].\;\;\;\;\; \end{array}$$

These ten additional parameters we define as the *secondary* fitting parameters. The statistical meaning of these parameters are the following. The parameters *A_H, mn_* characterize the amplitudes of beta-distributions referring, correspondingly, to the heights (*H*) and mean values (*mn*). The sum (α + β)_*H, mn*_ contains the information about their power-law exponents, *w*_1, *H, mn*_ gives the information about their asymmetry, max(*Bd_H_*, *Bd_mn_*) signifies their heights, and the fifth parameter SRA_*H, mn*_ contains information about the mean values of these two additional distributions.

From our point of view, these 20 (10 primary and 10 secondary) significant parameters [figuring in Expressions (14)–(16)] combined together can completely characterize the behavior of fluctuations associated with the long-time series analyzed and containing *N*_*total*_ = 2.5.10^5^ ÷ 10^6^ and even more data points.

### Clusterization of final parameters based on the generalized pearson correlation function

For clusterization purposes one can suggest more accurate selection of similar sequences based on *internal* correlations. For this aim we introduce the generalized Pearson correlation function (GPCF) (Nigmatullin, 2010; Nigmatullin et al., 2012).

(17) $$GPCF_{p}=\frac{GMV_{p}(s_{1},s_{2})}{\sqrt{GMV_{p}(s_{1},s_{1})\cdot GMV_{p}(s_{2},s_{2})}},$$

where expression:

(18) $$\begin{array}{l} GMV_{p}(s_{1},s_{2},\ldots ,s_{K})=\\ {\left(\frac{1}{N}\sum_{j\;=\;1}^{N}{\left|nrm_{j}(s_{1})\cdot nrm_{j}(s_{2})\cdot \ldots \cdot nrm_{j}(s_{K})\right|}^{mom_{p}}\right)}^{1/mom_{p}},\;\;\;\;\;\; \end{array}$$

determines the generalized mean value (*GMV*)-function of the *K*-th order. Here the generalized mean value (GMV) function determines the mean value for all range of the moments (see Expression (19) below). The set of parameters (*s*_1_,*s*_2_,…,*s_K_*) determines the type of the random sequence compared. The *GPCF_p_* determined by Expression (17) coincides with the conventional definition of the Pearson correlation coefficient at *mom*_p_ = 1. The set of moments are determined by the following expression:

(19) $$\begin{array}{l} mom_{p}=\exp(Ln_{p}),\;Ln_{p}=mn+\left(\frac{p}{P}\right)\cdot (mx-mn),\\ \;p=0,1,\ldots ,P. \end{array}$$

The value *mom_p_* in (19) corresponds to the current moment from the interval [0, *P*]. The value *P* determines the final value of the linear function *Ln_p_* located in the interval [*mn*, *mx*]. The values *mn* and *mx* define correspondingly the limits of the moments in the uniform logarithmic scale. In many practical cases these values are chosen as *mn* = −15, *mx* = 15 and *P* is chosen as an integer value located in the interval [50 ÷ 100]. This empirical choice is related to the fact that the transition region of the random sequences considered and expressed in the form of the *GMV*-functions is concentrated usually in the interval *Ln_p_* ∈ [−5, 5]. The extended interval [−15, 15] is taken usually for calculation of the limiting values of this function in the space of the fractional moments. The initial sequences are chosen in that way: the minimum of the GMV-function coincides with zero value while the upper value of this function coincides with the maximal value of the random sequence considered. In formula (18) the random sequence is normalized to the unit value in accordance with Expressions (*A*) and (*B*):

(20a) $$\begin{array}{l} (A)nrm_{j}(y)=\frac{y_{j}^{\left(+\right)}}{\max(y_{j}^{\left(+\right)})}-\frac{y_{j}^{\left(-\right)}}{\min(y_{j}^{\left(-\right)})},\\ \;y_{j}^{\left(\pm \right)}=\frac{1}{2}\left(y_{j}\pm \left|y_{j}\right|\right), \end{array}$$

(20b) $$\begin{array}{l} (B)nrm_{j}(y)=\frac{\Delta y_{j}}{\max(\Delta y_{j})},\Delta y_{j}=y_{j}-\min(y_{j}).\\ \;j=1,\;2,\ldots ,N,\;0<nrm(y)<1.\; \end{array}$$

Here, as it was done above, the set *y_j_* defines an initial random sequence that can contain a trend or can be compared with another trendless sequence. The symbol | … | and index *j* (*j* = 1.2,…,*N*) determine the absolute value and number of the measured points, correspondingly. The second case (*B*) in [20(b)] corresponds to the case when the initial sequence is positive. If the limits *mn* and *mx* in (20) have opposite signs and accept sufficiently large values, then the GPCF function has two plateaus (equaled unit at small numbers of *mn* (i.e., *GPCF_mn_* = 1) and another limiting value *GPCF_mx_* depends on the degree of internal correlation between two random sequences compared. This right-hand limit (defined as *Lm*) is located between two values:

(21) $$M\equiv \min(GPCF_{p})\leq Lm\equiv GPCF_{mx}\leq 1.$$

The appearance of two plateaus implies that all information about possible correlations is complete and further increasing of the limiting numbers (*mx*, *mn*) figuring in (19) is *useless*. Numerous tests showed that the high degree of correlations between two random sequences is achieved when *Lm* = 1, while the lowest correlations are observed when *Lm* = *M*. This empirical observation, having a general character for all random sequences, allows us to introduce new correlation parameter *CC* (complete correlation)—factor, which is determined as:

(22) $$CC=M\cdot \left(\frac{Lm-M}{1-M}\right).$$

We would like to stress here that this factor is determined on the *total* set of the fractional moments located between exp(*mn*) and exp(*mx*). As it was mentioned above, in practical calculations for many cases it is sufficient to put *mn* = −15 and *mx* = +15. The CC-factor accepts the unit values when the degree of correlation is high while the case *Lm* = *M* corresponds to the lowest (remnant) degree of correlations that can be observed between the compared random sequences. In addition, we want to stress also the following fact. This CC-factor does *not* depend on the amplitudes of the random sequences. The pair random sequences compared should be normalized to the interval: 0 ≤ |*y_j_*| ≤ 1. It reflects the *internal* structure of correlations of the compared random sequences based presumably on the similarity of their probability distribution functions that are *not* known in many cases. Recent example related to application of the statistics of the fractional moments was considered in paper (Nigmatullin et al., 2012). So, the CC-factor (22) can be used for clusterization of the significant parameters based on the following idea. For a set of significant parameters referring to one qualitative factor one can calculate the limits of CC-factor:

(23) $$cf_{\min}\leq CC\leq 1.$$

Here the low correlation limit *cf_min_* is determined by the sampling volume and conditions of experiment that should be almost the same for two qualitative factors compared (control/influence of another qualitative factor).

## Results

### Processing of the long-time membrane current series

In previous Section we described in details (**S1**–**S5**) basic steps of treatment of an arbitrary long-time series. Here we want to make some general remarks related to this procedure. If the long-time series considered contains the clearly expressed but random trend then its random behavior can disturb the monotonic behavior of the primary 9 parameters figuring in the fitting functions (15) and (16). In this cases one can recommend to apply the POLS (procedure of the optimal linear smoothing) described in papers (Baleanu et al., 2011; Ciurea et al., 2011; Nigmatullin et al., 2012) or simple numeric differentiation. These two procedures help to suppress the hidden random trend and obtain the monotonic behavior for the 9 parameters figuring in (15) and (16). In the shown figures we used the scaling factor ξ = 2. For the rational values of ξ from the interval (1, 2) Expression (9) can be modified as:

(24) $$\Delta_{k}=\frac{N_{tot}}{\exp\left[\left(K+1-k\right)\ln(2)\cdot \mu \right]},\;\mu =\frac{\ln\left(\xi \right)}{\ln\left(2\right)}.$$

So, numerical calculations realized at ξ = 1.5 show that results are *not* changed essentially, only the integer variable *k* in Expressions (15) and (16) is replaced as *k*→ μ*k*. We think that this method has a wide range of its applicability and these two modifications can be taken into account in order to express the long-range time series in terms of 20 significant parameters. In similar manner as it was treated the membrane currents for the randomly taken interneuron-3 one can treat other long-time series related to other (1, 2, 4, 5, 6, 7) interneurons. Besides, in order to differentiate these random sequences recorded *without* presence of a biological object we treated in the same manner 6 random sequences corresponding to empty electrode.

The next problem is associated with the finding of criterion of clusterization that helps to combine these “control” membrane currents to one strongly-correlated cluster based on the values of the significant parameters. For each cell these parameters are collected in Table 1. For 6 files corresponding to pure solute (without presence of the cell) the results are collected in Table 2. How to differentiate these 20 quantitative parameters (in this case a qualitative factor is associated with the presence/absence of a biological cell) from each other? The simplest classification can be related to calculation of the mean value and standard deviation of the calculated significant parameter in each row. But more effective scheme of clusterization based on the statistics of the fractional moments and the usage of the complete correlation factor is considered in the next section.

**Table 1:** **The collection of 20 significant parameters calculated for 7 cells based on calculation of registered membrane currents**.

**Parameter**	**Cell-1**	**Cell-2**	**Cell-3**	**Cell-4**	**Cell-5**	**Cell-6**	**Cell-7**
max(*w*_1_)	**0.4997**	0.5006	**0.5027**	0.5027	0.5019	0.5006	0.5003
λ*_a_*	−0.3605	**−0.3613**	**−0.3517**	−0.3571	−0.3586	−0.3581	−0.3604
*A*_1_	0.5719	0.5943	**1.2790**	**0.5683**	0.6528	0.8815	0.8768
*A*_0_	−0.00149	−0.00149	**−0.00328**	**−0.00147**	−0.00167	−0.00224	−0.00219
ν	0.995	0.995	0.995	0.995	0.995	0.995	0.995
*A_pl_*	**1.558**	1.558	**1.544**	1.552	1.554	1.553	1.556
*B_pl_*	0.1308	0.1295	**0.1374**	**0.1277**	0.1306	0.1315	0.129
λ*_B_*	**0.6666**	0.6650	0.6646	0.6654	0.6641	**0.6640**	0.6656
*B*_1_	**−0.2111**	−0.2418	**−0.4333**	−0.2266	−0.2438	−0.3318	−0.3959
*B*_0_	**7.807**	8.801	**15.60**	8.247	8.717	11.89	14.56
*A_H_*	0.0292	0.0314	**0.0708**	**0.0265**	0.0379	0.0653	0.0440
(α + β)*_H_*	1.604	1.598	1.596	**1.613**	1.589	**1.560**	1.606
max(*w*_1_*_H_*)	0.5208	**0.5136**	0.5158	0.5233	0.5160	**0.5257**	0.5174
max(*Bd_H_*)	18580	18980	**42070**	**18110**	21110	29110	28300
mn(SRA*_H_*)	35.68	37.01	**78.46**	**34.87**	40.42	54.57	54.30
*A_mn_*	0.00212	**0.00107**	**0.00303**	0.00123	0.00139	0.00242	0.00134
(α + β)*_mn_*	**1.517**	1.57	1.538	1.576	1.552	1.53	**1.592**
max(*w*_1_*_mn_*)	0.5003	0.4997	**0.4954**	0.5011	**0.5013**	0.4991	0.5
max(*Bd_mn_*)	650.4	**506.9**	**1107.0**	614.5	570.7	837.3	766.1
mn(*SRA_mn_*)	4.986·10^−4^	2.277·10^−4^	**0.00304**	2.205·10^−4^	**−0.00157**	6.655·10^−4^	0.00258

*Each column describing the chosen cell is obtained in the result of the averaging of three membrane currents with the length 250,000 data points. The first 10 primary parameters are marked by a double line. The minimal and maximal values of each significant parameter in each row are bolded*.

**Table 2:** **The collection of 20 significant parameters calculated for 6 files corresponding to currents recorded with the empty electrode placed inside an artificial cerebrospinal fluid (the biological material is absent)**.

**Parameter**	**File-1**	**File-2**	**File-3**	**File-4**	**File-5**	**File-6**
max(*w*_1_)	0.5007	0.5029	0.4994	0.501	**0.4993**	**0.5032**
λ*_a_*	−**0.3567**	−**0.3703**	−0.3576	−0.3623	−0.3674	−0.3606
*A*_1_	5.455·10^−9^	5.488·10^−9^	5.457·10^−9^	**5.502·10^−9^**	**5.417·10^−9^**	5.441·10^−9^
*A*_0_	−**2.555·10^−11^**	−**1.282·10^−11^**	−1.47·10^−11^	−1.413·10^−11^	−1.358·10^−11^	−1.423·10^−11^
ν	0.995	0.995	0.995	0.995	0.995	0.995
*A_pl_*	1.562	**1.569**	1.557	1.562	**1.569**	**1.560**
*B_pl_*	0.1242	**0.1278**	0.1315	0.1290	0.1294	**0.1320**
λ*_B_*	**0.6395**	0.6506	0.6859	0.6398	0.6506	**0.6944**
*B*_1_	**−3.349·10^−9^**	−2.479·10^−9^	−1.986·10^−9^	−3.336·10^−9^	−2.479·10^−9^	**−1.649·10^−9^**
*B*_0_	**1.023·10^−7^**	**7.672·10^−8^**	9.194·10^−8^	9.272·10^−8^	7.675·10^−8^	8.584·10^−8^
*A_H_*	3.202·10^−10^	2.211·10^−10^	**6.638·10^−12^**	3.232·10^−10^	2.231·10^−10^	**3.238·10^−10^**
(α + β)*_H_*	1.591	1.637	**1.585**	**1.721**	1.631	1.592
max(*w*_1_*_H_*)	0.5104	0.5136	**0.4986**	**0.5704**	0.5436	0.5131
max(*Bd_H_*)	1.820·10^−4^	1.847·10^−4^	**3.583·10^−6^**	**2.920·10^−4^**	1.907·10^−4^	1.853·10^−4^
mn(SRA*_H_*)	**3.471·10^−7^**	3.469·10^−7^	**2.775·10^−13^**	3.171·10^−7^	3.369·10^−7^	3.448·10^−7^
*A_mn_*	**6.094·10^−12^**	**8.315·10^−12^**	6.638·10^−12^	6.014·10^−12^	8.227·10^−12^	6.731·10^−12^
(α + β)*_mn_*	**1.600**	1.563	1.585	1.556	1.569	**1.524**
max(*w*_1_*_mn_*)	0.5009	**0.4964**	0.4986	0.5109	0.5064	**0.5169**
max(*Bd_mn_*)	**3.733·10^−6^**	3.709·10^−6^	3.583·10^−6^	**3.233·10^−6^**	3.711·10^−6^	3.385·10^−6^
mn(*SRA_mn_*)	**1.064·10^−11^**	7.818·10^−12^	2.775·10^−13^	1.004·10^−11^	7.821·10^−12^	**2.557·10^−13^**

*The first 10 primary parameters are marked by a double line. The minimal and maximal values of each significant parameter in each row are bolded*.

For the clusterization of final parameters we have used new correlation parameter *CC* described in “Materials and Methods” section Expression (22). The calculation of the CC-factor (in our case it is based on a set of membrane currents associated with 3 “control” measurements for each chosen cell from the total set of currents representing other 7 biologic cells) which is considered as the complex correlation matrix (see Table 3) having minimal dimension (7 × 7) leads to the minimal value *cf_min_* = 0.9238. The result is not changed essentially if one calculates numerically the corresponding integrals with respect to their normalized significant parameters and then considers their CC-factors. The tendency of the strong correlations between columns of Table 1 is conserved, only the boundary of the correlation interval is slightly increased achieving the value *Jcf_min_* = 0.9736. So, using the method of clusterization based on the statistics of the fractional moments and Expression (22) one can say that all “control” currents measured for the sampling 7 × 7 = 49 form the strongly-correlated cluster with limits [0.9238, 1] for the initial set of significant parameters (20 parameters for each sampling) and [0.9736, 1] (for the corresponding integrals that are obtained by direct trapezoid method from the normalized significant parameters). In accordance with this method of clusterization one can make the following conclusion: if any another series having 20 significant parameters will give the CC-factor located in the interval [0.9238, 1] then it can be considered as the “friend” file belonging to this cluster, in the opposite case it can be considered as a “strange” file. For more reliable identification the saying above can be referred to the integrated columns formed from 20 normalized significant parameters. In the same manner we treated the files corresponding to the electrode currents recorded in normal saline solution without presence of biological object. The 20 desired parameters for 6 files are collected in Table 2. Their correlation matrix presented by Table 4 form another cluster. But attempt to combine the currents corresponding to the living o cells with currents corresponding to empty electrodes located in saline solution is *unsuccessful*. If we compare the correlation matrix of Table 5 with the previous ones (Tables 3, 4) then one can notice that the last matrix is *uncorrelated* (all elements are close to zero). It means that the presence of the biologic cell completely changes the statistical structure of the current and from qualitative point of view the long-time random sequences of currents recorded for both cases (presence/absence of biological cell) are *different*.

**Table 3:** **The correlation matrix of the calculated CC-factors [Expression (22)] for 20 parameters characterizing 7 neurons collected in the Table 1**.

**Cells**	**Cell-1**	**Cell-2**	**Cell-3**	**Cell-4**	**Cell-5**	**Cell-6**	**Cell-7**
Cell-1	1	0.99876	0.92838	**0.99957**	0.99841	0.9767	**0.94824**
Cell-2	0.99876	1	**0.93615**	0.99954	**0.99981**	0.98465	0.95698
Cell-3	0.92838	0.93615	1	**0.93354**	0.93708	0.97193	**0.99714**
Cell-4	0.99957	0.99954	0.93354	1	**0.99933**	0.98166	**0.95451**
Cell-5	0.99841	0.99981	0.93708	0.99933	1	**0.98558**	**0.95776**
Cell-6	0.9767	0.98465	0.97193	0.98166	0.98558	1	0.9804
Cell-7	0.94824	0.95698	0.99714	0.95451	0.95776	0.9804	1

*The maximal and minimal values of correlations in each row are bolded*.

**Table 4:** **The correlation matrix of the calculated CC-factors for 20 parameters characterizing 6 empty electrode records collected in the Table 2**.

**Files**	**F-1**	**F-2**	**F-3**	**F-4**	**F-5**	**F-6**
F-1	1	0.99581	**0.97238**	0.99076	**0.99642**	0.99624
F-2	0.99581	1	**0.97241**	**0.99767**	0.99995	0.99951
F-3	0.97238	0.97241	1	**0.97008**	**0.97244**	0.97237
F-4	0.99076	0.99767	0.97008	1	**0.99706**	**0.99588**
F-5	0.99642	0.99995	0.97244	0.99706	1	0.9996
F-6	0.99624	0.9995	0.97237	0.99588	0.9996	1

*The maximal and minimal values of correlations in each row are bolded*.

**Table 5:** **The correlation matrix of the CC-factors calculated for 7 cells and 6 empty electrodes**.

**Cells\ Files**	**F-1**	**F-2**	**F-3**	**F-4**	**F-5**	**F-6**
Cell-1	0.01809	0.01807	0.01387	0.01883	0.01805	0.01781
Cell-2	0.01772	0.01769	0.01357	0.01844	0.01767	0.01743
Cell-3	0.00889	0.00887	0.00666	0.00929	0.00886	0.00873
Cell-4	0.01807	0.01805	0.01386	0.0188	0.01802	0.01778
Cell-5	0.01679	0.01676	0.01282	0.01748	0.01674	0.01652
Cell-6	0.01343	0.01341	0.01018	0.014	0.01339	0.0132
Cell-7	0.01212	0.0121	0.00918	0.01264	0.01208	0.01191

So, new clusterization method helps to express quantitatively the internal factor as the presence/absence of the living cell (compare this statement with series shown on Figure 1 where the corresponding currents look similar to each other). Definitely, more accurate measurements are needed in order to differentiate from many mixed factors that form a time-series for biological and non-biological objects a specific *predominant* factor that plays an essential role in this differentiation. But this problem merits a separate research.

## Discussion

It is well known that cellular membrane is the element which largely provides cell functioning. Cell membrane has so many functions that it is difficult even to list—anyone can find them all in each textbook on cell biology. In general the membrane provides all interaction of the cell with the external environment including the perception of the effect of active substances. Withal the membrane comprises a lot of elements which produce so-called “membrane noise”—rather small variations of membrane potential or trans-membrane current; mainly they are different types of ion channels, transporters and pumps. There are many active substances affecting the operation of these elements so the action of these substances actually can be detected by analyzing the membrane noise. But even if some substance does not affect channels, transporters or pumps directly its action often can be detected by noise analysis too. For example if the substance affects G protein-coupled receptors or state of membrane lipids—in many cases it leads to the changes in the functioning of ion channels (Tillman and Cascio, 2003; Inanobe and Kurachi, 2014) and, accordingly, to the noise changes. So the analysis of the long-time series of noise can help to detect the action of many substances when we cannot detect this action differently.

For analysis of the long-time series we applied new BRC method based on the beta-distribution function. Four parameters of the beta-distribution function can be used for description of the local fluctuations and the averaged beta-distributions can be applied for *quantitative* reading of series containing large number of data points. The fluctuation spectroscopy based on beta distribution allows realizing the essential reduction (2.5–10).10^5^ data points to 20 quantitative parameters *only* [see Expressions (14)–(16)] that contain the basic information calculated from three basic beta-distributions: (1) distribution over different segments (scales), (2) the secondary beta-distributions over their heights and (3) distributions over mean values. This reduction becomes possible thanks to the invariant properties that are expressed by formulae (3) and (5). We suppose that this approach can be applied successfully for the unified additional analysis of fluctuations of different long-time series that present the results of monitoring of biological, medical and other data reflecting the results of response of the complex system considered with respect to some external factor. In particular, this BRC method is applicable to testing the action of antagonist of receptor and ion channels when the modification based on different type of interaction (with binding site or with the open channel with different kinetics). In such experiments in order to understand the mechanism of action of some new substances we only need to compare the FSBD parameter changes caused by this substance with typical changes stored in the database.

## Funding

This work was partially supported (Andrei I. Skorinkin) by RF grant “Leading Scientific School” and RFBR grant.

### Conflict of interest statement

The authors declare that the research was conducted in the absence of any commercial or financial relationships that could be construed as a potential conflict of interest.
